# Inhibitors of PD-1/PD-L1 and ERK1/2 impede the proliferation of receptor positive and triple-negative breast cancer cell lines

**DOI:** 10.1007/s00432-021-03694-4

**Published:** 2021-06-29

**Authors:** Karen Bräutigam, Elodie Kabore-Wolff, Ahmad Fawzi Hussain, Stephan Polack, Achim Rody, Lars Hanker, Frank Köster

**Affiliations:** 1 Department of Gynecology and Obstetrics, University Medical Center Schleswig-Holstein, Campus Lübeck, Lübeck, Germany; 2 Department of Gynecology and Obstetrics, Medical Faculty, Justus-Liebig-University Giessen, Giessen, Germany

**Keywords:** Triple-negative breast cancer, Immune checkpoint inhibitor, ERK inhibitor, Proliferation, Combined therapy

## Abstract

**Purpose:**

Triple-negative breast cancer (TNBC) is characterized by an unfavorable prognosis and missing systemic therapeutic approaches beside chemotherapy. Targeting the immune checkpoint PD-1/PD-L1 showed promising results in breast cancer and especially in TNBC. The extracellular signal-regulated kinase 1/2 (ERK1/2) is an important driver of carcinogenesis. Here, the effect of combined PD-1/PD-L1 and ERK1/2 inhibitor treatment is investigated of cell growth and intracellular impact of breast cancer cell lines.

**Methods:**

The IC_50_ values of each inhibitor and the effect of combined treatment were determined in three TNBC cell lines of different subtypes and one non-TNBC cell line. Phospho-specific antibodies were used in western blot analyses to investigate an effect on ERK1/2 activation. Expressions of immune modulatory and cell cycle-associated genes were examined by quantitative reverse transcription PCR.

**Results:**

Both inhibitors PD-1/PD-L1 and ERK1/2 impeded the proliferation of TNBC to a higher extent than of non-TNBC. By combined treatment, cell lines were inhibited either synergistically or additively. ERK1/2 and S6 phosphorylation were reduced and expressions of c-Fos and FosL were diminished after ERK1/2 inhibitor as single and combined treatment. Between genes involved in immune modulation, IL-8 was upregulated in TNBC cells after combined treatment.

**Conclusion:**

In conclusion, combination of PD-1/PD-L1 and ERK1/2 inhibitors showed favorable effects for a new therapy strategy, with better results in TNBC cell lines than in non-TNBC cells. The effects have to be validated in models that can reflect the interaction between immune and tumor cells like the situation in the tumor micro-environment.

## Introduction

Breast cancer is the most common cancer among women in Germany and worldwide (Siegel et al. 2019). 80–85% of breast cancers display hormone receptor expression and/or overexpression of Her-2 receptor (Cancer Genome Atlas 2012). Receptor-positive breast cancer subtypes are predictive for endocrine therapy and Her-2 receptor-targeted antibody therapy. 15–20% of breast cancer cells express none of these receptors and are called triple-negative breast cancer (TNBC). TNBC is associated with higher recurrence and metastasis rates and lower overall survival. Furthermore, the treatment options besides chemotherapy are limited (Dent et al. 2007). TNBCs are a heterogeneous group subdivided into 4 subtypes: basal-like1 (BL1), basal-like2 (BL2), mesenchymal (M) and luminal androgen receptor (LAR) subtype (Lehmann et al. 2016).

The mitogen-activated protein kinases (MAPK) pathways are intracellular signal cascades that regulate multiple cell processes by consecutive phosphorylation of signal transduction proteins. The RAS/RAF/MEK/ERK pathway is the classical MAPK pathway activated by growth factors, cytokines, integrins as well as steroids. The MAPK pathway regulates cell proliferation, migration and angiogenesis (Santen et al. 2002). Several studies used small molecule drugs to inhibit the MAPK pathway in various tumor entities, but results for specific ERK1/2 inhibitors in breast cancer are rare.

The selective ERK inhibitor SCH772984, used in this study, not only impedes dose-dependently the phosphorylation of ERK, but also the phosphorylation of S6, one of its downstream targets (Meyuhas 2015).

Besides the MAPK pathway, the STAT3 (signal transducer and activator of transcription 3)-pathway plays an important role in progression and metastasis of breast cancer by promoting angiogenesis and is increasingly phosphorylated in ca. 40% of breast cancers (Chang et al. 2013).

PD-1 (programmed cell death protein 1) is a transmembrane receptor expressed on immune cells representing an immune checkpoint (Arasanz et al. 2017). PD-1 interacts with two ligands, PD-L1 and 2 (programmed cell death 1 ligand 1 and 2). PD-L1 expression is increased on breast cancer cells and is correlated with tumors having a low prognosis. PD-L1 is more abundant in the subgroup of TNBC (Gatalica et al. 2014; Migali et al. 2016) and expressed in 20–30% of TNBC (Nanda et al. 2016). PD-L1 modulates the immune response (Patel and Kurzrock 2015). The interaction of PD-L1 with PD-1 inhibits the activation of T lymphocytes and the proliferation of regulatory T cells infiltrating the tumor and thereby prevents the immune response (tumor immune escape) (Francisco et al. 2010). Many clinical studies described overexpression of PD-L1 in different tumor entities and the anti-tumoral effect by the inhibition of PD-1/PD-L1 interaction. By now, several immune checkpoint inhibitors are applied in the guidelines of therapy regimes for different cancer types, e.g. melanoma and small cell lung cancer. For TNBC, a study of 902 patients showed an overall advantage for patients treated with a combination of the anti-PD-L1 antibody atezolizumab and nab-paclitaxel compared to a therapy with only nab-paclitaxel (Schmid et al. 2020). Atezolizumab is approved for therapy of non-resectable, advanced or metastasized TNBC with PD-L1 positivity in ≥ 1% of tumor surrounding immune cells (Schmid et al. 2020). Solinas and colleagues described positive effects of combination of PD-1/PD-L1 inhibitors in combination with established therapeutics in breast cancer and especially in TNBC (Solinas et al. 2017). Dependent on the type of cancer, the PD-L1 expression is differently regulated, e. g. in lymphoma and lung cancer PD-L1 expression is elevated by the ALK (anaplastic lymphoma kinase) through STAT3 activation (Marzec et al. 2008). Cui and coworkers described that PD-L1 expression influences EMT (epithelial mesenchymal transition) in oral squamous cell carcinoma. In oral squamous cell carcinoma cell lines, PD-L1 is regulated by ERK and STAT3 signaling. Downregulation of PD-L1 with siRNA was accompanied by downregulation of mesenchymal proteins and decreased phosphorylation of ERK and STAT3 (Cui et al. 2021). Also, ERK mediates PD-L1 expression in NSCLC (non small cell lung carcinoma) cells with ROS1 fusion: Liu and coworker showed that both ERK phosphorylation and PD-L1 expression were down-regulated after treating HCC78 cells with PD0325901 or U0126 (Liu et al. 2020).

The aim of this study was to investigate the potential impact of either a PD-1/PD-L1 inhibitor or an ERK1/2 inhibitor and the combination of these in TNBC and non-TNBC cell lines.

## Materials and methods

### Breast cancer cell lines

Four established breast cancer cell lines comprising three TNBC: MDA-MB-231 (M); HCC1937 (BL1); HCC1806 (BL2) and one non-TNBC (MCF7) were actually purchased from the American Type Cell Collection (LGC, Wesel, Germany). Cells were cultivated in Dulbecco's modified Eagle's medium (DMEM)) containing 10% fetal bovine serum, 1% penicillin–streptomycin (100x) (all from Life Technologies, Darmstadt, Germany) and incubated at 37 °C in a humidified atmosphere with 5% CO_2_.

### Compounds

PD-1/PD-L1 inhibitor 1 (BMS-1) and ERK1/2 inhibitor (SCH772984) were purchased from Selleckchem (Absource Diagnostics GmbH, Munich, Germany) and diluted in dimethyl sulfoxide (DMSO) at 10 mM and 8.51 mM, respectively.

### Cell proliferation assay

Cell proliferation was determined by the MTS assay (CellTiter 96^®^ AQueous One Solution Reagent, Promega, Walldorf, Germany). A total of 1–2 × 10^3^ cells were plated in 100 µl medium in each well of a 96-well plate. After 24 h, PD-1/PD-L1 and ERK1/2 inhibitor were diluted to the desired concentrations in test medium (DMEM containing 1% fetal bovine serum) and added as single or as combined treatment in triplicate for 72 h. The MTS assay was repeated at least three times for each cell line to determine the half maximum inhibitory concentration (IC_50_) and an interaction index.

### Western blot

4 × 10^5^ cells were seeded onto a 6-well plate for 24 h and treated for further 24 h. For protein isolation 100 µl RIPA buffer (phosphate-buffered saline, 1% Igepal, 0.5% sodium deoxycholate, 0.1% SDS) with freshly added 1% protease inhibitor and phosphatase inhibitor 2; (Sigma-Aldrich, Hamburg, Germany) was used. 20 µg per sample were transferred onto polyvinylidene difluoride membranes (Carl Roth, Karlsruhe, Germany). The immune analyses were performed with antibodies in the following mixing ratio: Phospho-p44/42 MAPK (ERK1/2) (1:2,000); p44/42 MAPK (ERK1/2) (1:1,000); Phospho-S6 Ribosomal Protein (1:2,000); S6 Ribosomal Protein (1:1,000); Phospho-STAT3 (1:2,000); STAT3 (1:1,000) (all from Cell Signaling Technology, Frankfurt am Main, Germany). For immune detection, we used the Clarity Max Western ECL Substrate (Biorad, Feldkirchen, Germany) and the immune reaction was visualized by the Chemidoc Imaging System and analyzed by Image Lab (Biorad).

### RNA isolation, reverse transcription and quantitative real-time polymerase chain reaction (qPCR)

4 × 10^5^ cells were grown and treated on a 6-well plate for 24 h and RNA was extracted using the RNeasy Mini Kit (Qiagen, Hilden, Germany). 1 µg RNA was reverse transcribed into cDNA using Superscript-II-Reverse-Transcription from Invitrogen (Fisher Scientific, Schwerte, Germany). QPCR was performed using GoTaq qPCR Master Mix (Promega) and the following primer sequences for analyzing expression of immune modulatory and growth-associated genes: PD-L1: forward 5ʹ-GGACAAGCAGTGACCATCAAG-3ʹ, reverse 5ʹ-CCCAGAATTACCAAGTGAGTCCT-3ʹ; interleukin 8 (IL-8): forward 5ʹ-ACTGAGAGTGATTGAGAGTGGAC-3ʹ, reverse 5ʹ-AACCCTCTGCACCCAGTTTTC-3ʹ; chemokine (C-X-C Motif) receptor 2 (CXCR2): forward 5ʹ-CCTGTCTTACTTTTCCGAAGGAC-3ʹ, reverse 5ʹ-TTGCTGTATTGTTGCCCATGT-3ʹ; c-Fos: forward 5ʹ-GAGATTGCCAACCTGCTGAA-3ʹ, reverse 5ʹ-AGACGAAGGAAGACGTGTAA-3ʹ, Fos-like 1 (FosL1): forward 5ʹ-CAGGCGGAGACTGACAAACTG-3ʹ, reverse 5ʹ-TCCTTCCGGGATTTTGCAGAT-3ʹ. PCR product specificity was verified by comparative melting curve analysis. Cycle threshold values of genes of interest were quantified, and normalized to expression of succinate dehydrogenase complex flavoprotein subunit A (SDHA) (forward: 5ʹ-TGGGAACAAGAGGGCATCTG-3ʹ, reverse: 5ʹ-CCACCACTGCATCAAATTCATG-3ʹ), and relative expression of genes in drug-treated cells was compared to relative expression of genes in untreated cells using the 2^−ΔΔCt^ method (Pfaffl 2001).

### Statistical analyses

All results were expressed as the mean and standard deviation (SD) from three independent experiments. The significance of the differences in cell proliferation using MTS assays after treatment between cell lines was examined in a Student’s two-tailed *t* test. The same test was performed to determine the significance of the differences of single and combined treatment with two inhibitors in MTS assays, western blots and qPCR. Statistical significance: *p < 0.05, **p < 0.01, ***p < 0.001.

## Results

### Influence of PD-1/PD-L1 inhibitor and ERK1/2 inhibitor on cell viability of TNBC and the non-TNBC cell lines

**Fig. 1:**
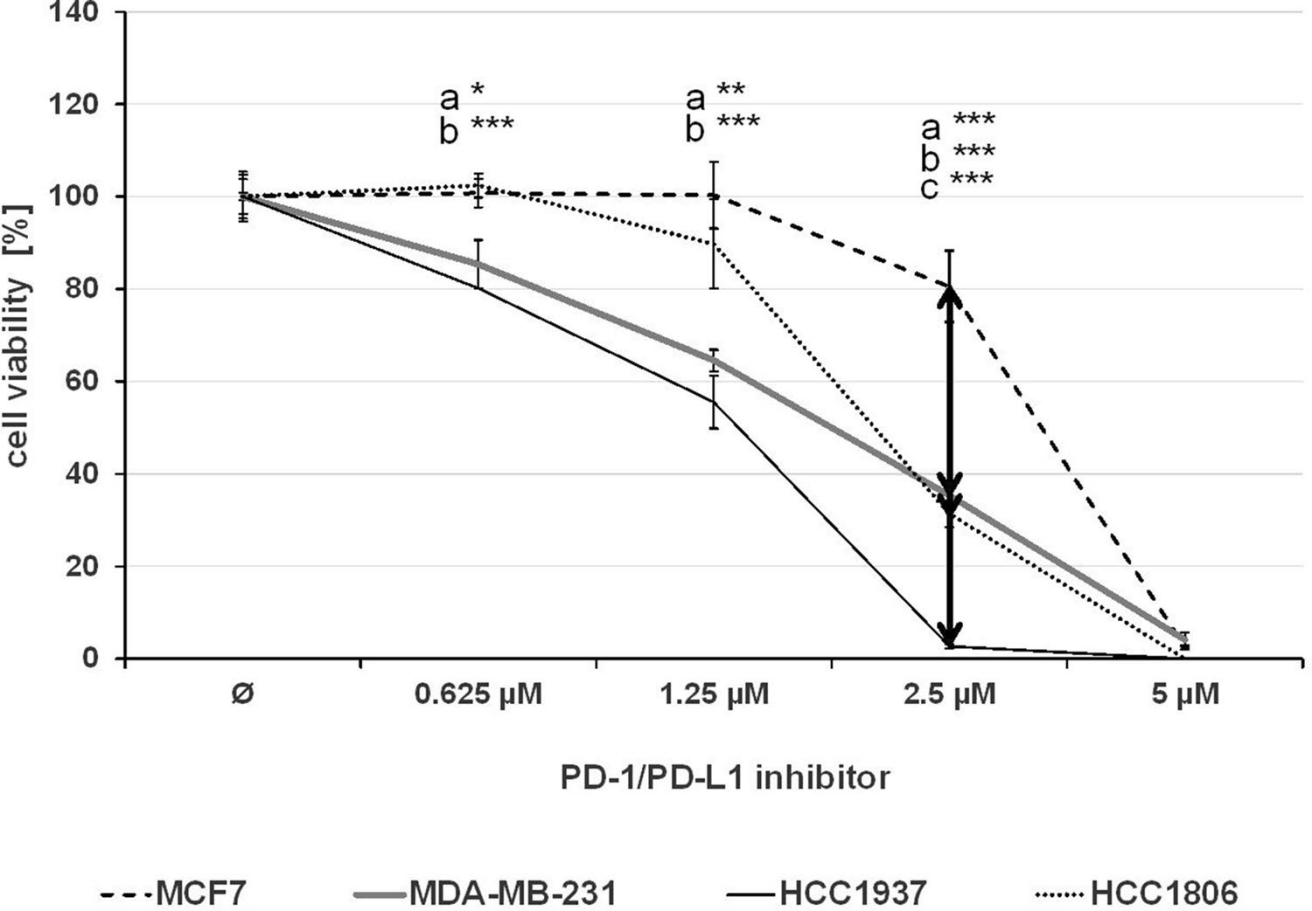
MTS assay after PD-1/PD-L1 inhibitor 1 treatment. The TNBC cells MDA-MB-231, HCC1937, HCC1806, and the non-TNBC cell line MCF7 were treated with rising concentrations of PD-1/PD-L1 inhibitor 1 for 72 h. Statistical significance of differences between MCF7 and **a** MDA-MB-231, **b** HCC1937, and **c** HCC1806: *p < 0.05, **p < 0.01, ***p < 0.001 (*t* test)

**Fig. 2:**
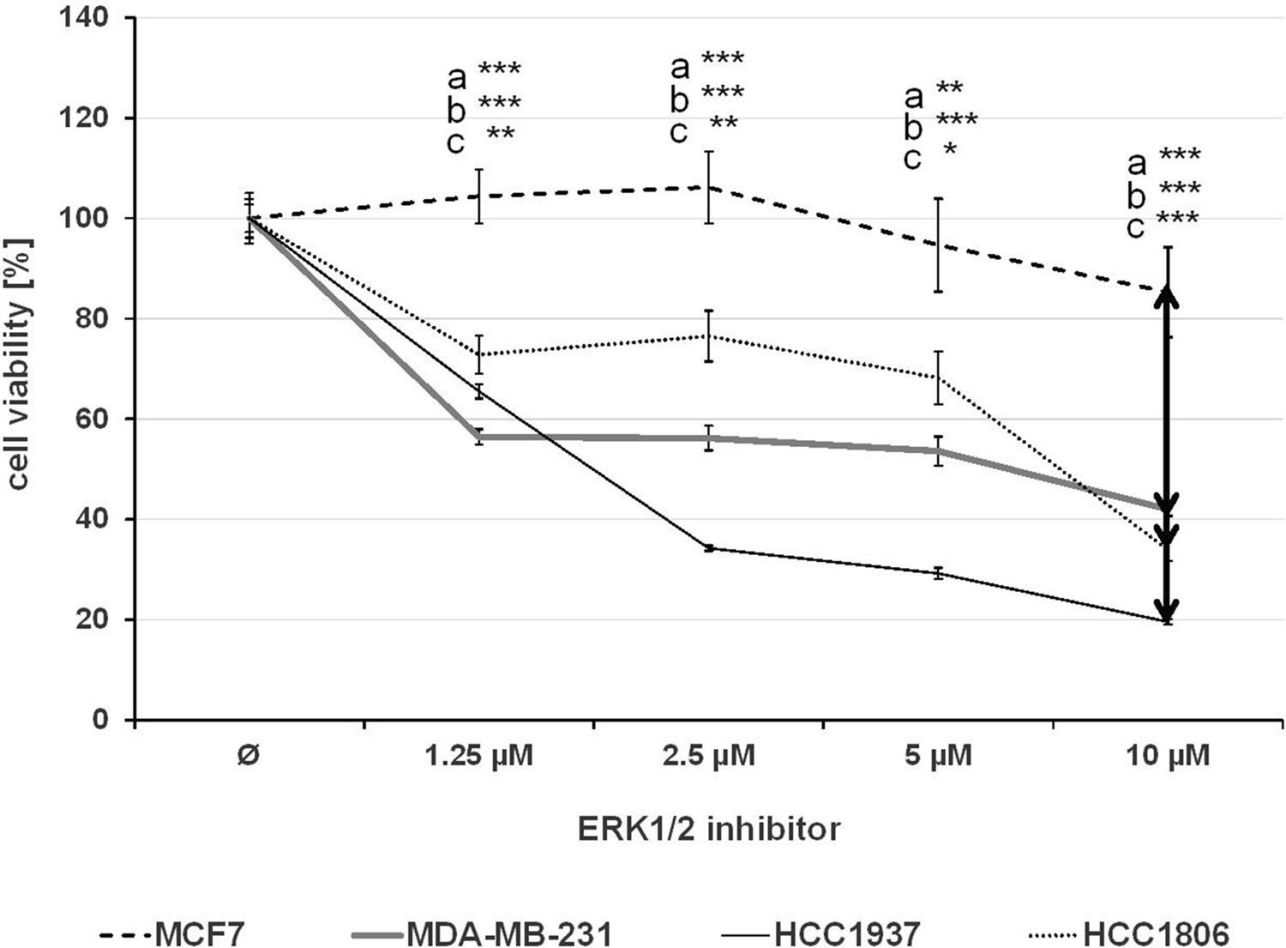
MTS assay after ERK1/2 inhibitor treatment. The TNBC cells MDA-MB-231, HCC1937, HCC1806, and the non-TNBC cell line MCF7 were treated with rising concentrations of ERK1/2 inhibitor for 72 h. Statistical significance of differences between MCF7 and **a** MDA-MB-231, **b** HCC1937, and **c** HCC1806: *p < 0.05, **p < 0.01, ***p < 0.001 (*t* test)

**Fig. 3:**
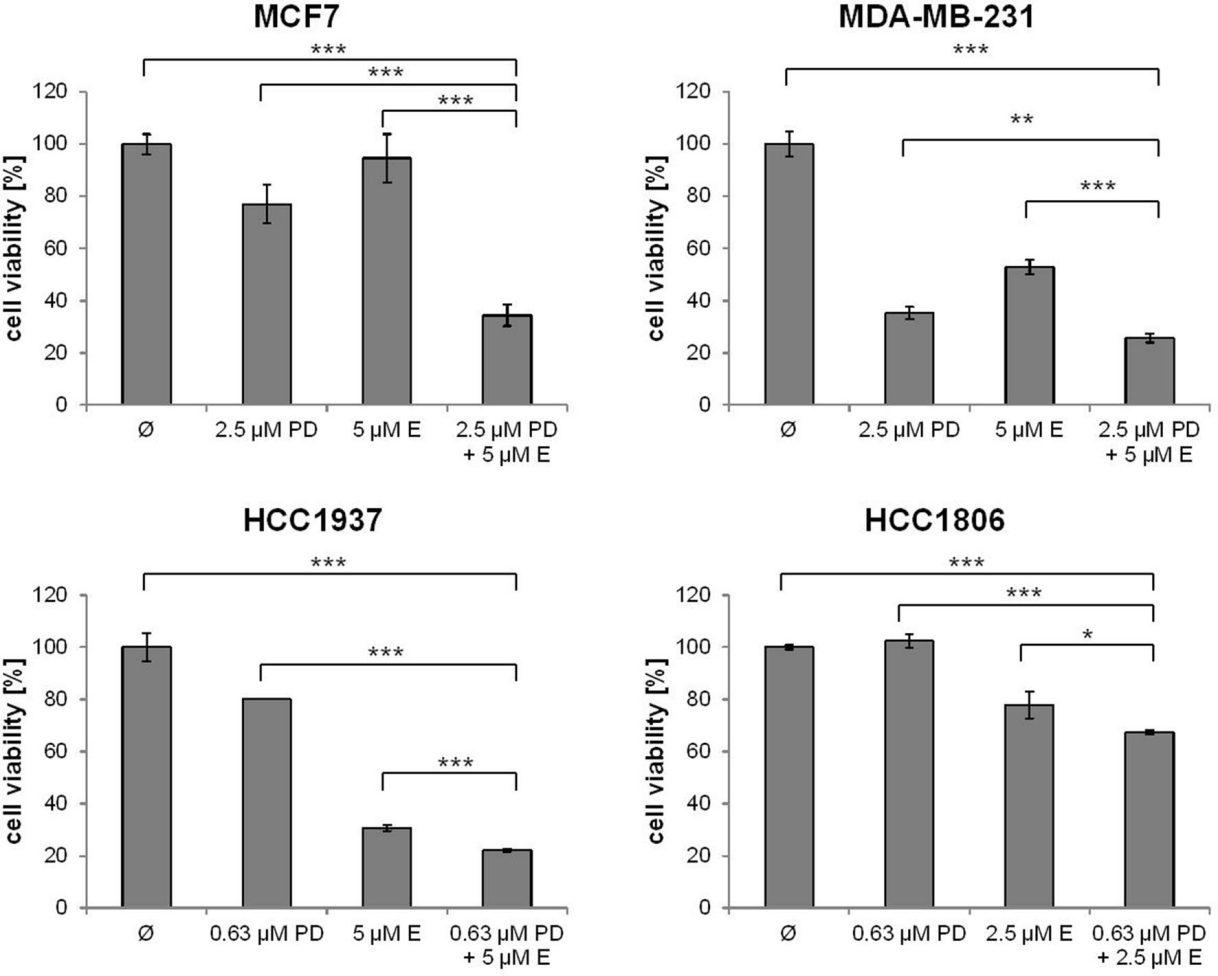
MTS assay after single and combined inhibitor treatment. The MDA-MB-231, HCC1937, HCC1806, and the MCF7 cells were treated with moderate concentrations of PD-1/PD-L1 inhibitor 1 (PD) and ERK1/2 inhibitor (E) apart and in combination (PD + E) for 72 h. Statistical significances between single and combined treatments: *p < 0.05, **p < 0.01, ***p < 0.001 (*t* test)

The effect of single treatment of the PD-1/PD-L1 inhibitor and the ERK1/2 inhibitor on cell viability was investigated in all cell lines after 72 h of treatment and the IC_50_ values were determined (Table 1). At a concentration of 2.5 µM, the PD-1/PD-L1 inhibitor significantly impeded the cell viability of TNBC cell lines MDA-MB-231, HCC1937 and HCC1806 to a greater extent compared to the non-TNBC cell line MCF7 (Fig. 1) The sensitivity towards the ERK1/2 inhibitor was significantly higher in the three TNBC cell lines compared with the non-TNBC cell line MCF7, which is shown by IC_50_ values in Table 1 and Fig. 2. Subsequently, the effect of combined treatment with adapted concentrations of the PD-1/PD-L1 inhibitor and the ERK1/2 inhibitor on cell viability was investigated to calculate the interaction index for both substances. The combination inhibited the proliferation in all cell lines stronger than single treatment (Fig. 3). The concurrent treatment led to an additive effect in the MDA-MB-231 cells (y = 1.02). In the basal-like cell lines HCC1937 (y = 0.88) and HCC1806 (y = 0.85) and the MCF7 (y = 0.76) cells, the cell viability was impeded synergistically (Table 2).

**Table 1:**
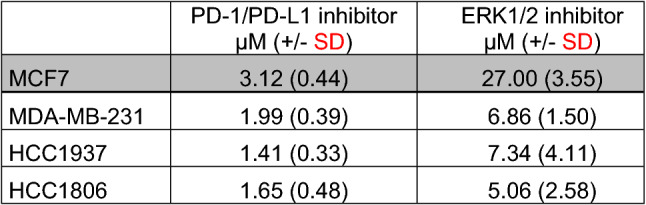
IC_50_ values estimated by MTS assays

IC_50_ values (µM) of the PD-1/PD-L1 inhibitor 1 and the ERK1/2 inhibitor for the TNBC cells MDA-MB-231, HCC1937, HCC1806 (white), and the non-TNBC cell line MCF7 (gray) were estimated and represented as the mean of three independently performed experiments

*SD* standard deviation

**Table 2:**
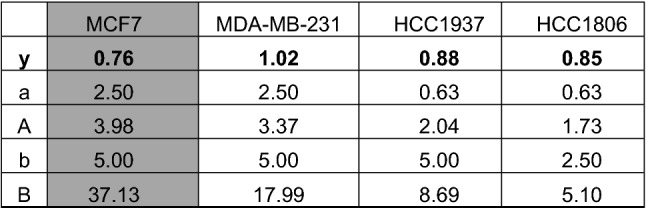
Calculation of interaction index values after combined treatment

The TNBC cells MDA-MB-231, HCC1937, HCC1806 (white), and the non-TNBC cell line MCF7 (gray) were treated simultaneously with moderate concentrations of the PD-1/PD-L1 inhibitor 1 (0.625 or 2.5 µM) and the ERK1/2 inhibitor (2.5 or 5.0 µM). The interaction indexes y (y = a/A + b/B) were calculated. Synergism: y < 1; additive effect: y = 1; antagonism: y > 1

### Impact of PD-1/PD-L1 inhibitor and ERK1/2 inhibitor on phosphorylation of ERK, S6 and STAT3

**Fig. 4:**
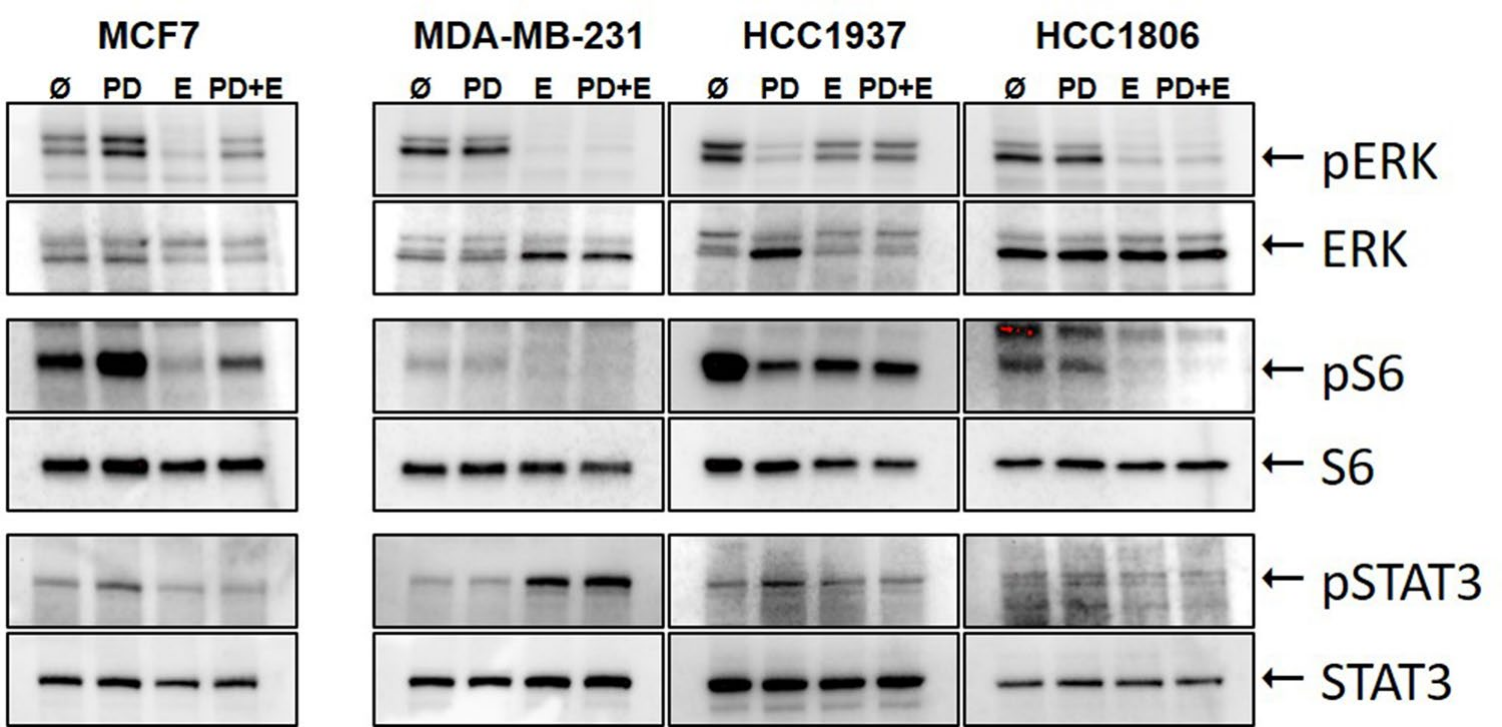
Western blots after single and combined inhibitor treatment. MCF7, MDA-MB-231, HCC1937, and HCC1806 cells were treated with 5 µM PD-1/PD-L1 inhibitor 1 (PD) and 5 µM ERK1/2 inhibitor (E) apart and in combination (PD + E) for 24 h and the phosphorylated and whole amount of ERK, S6, and STAT3 were detected in three independent experiments (one representative experiment is pictured)

TNBC and the non-TNBC cell lines were treated with the single inhibitors and their combination for 24 h and phosphorylations of ERK, S6 and STAT3 were determined by western blot analysis (Fig. 4). The percentage of phosphorylated to total protein was quantified considering protein loading amounts (Table 3). As expected, the ERK1/2 inhibitor caused a deactivation of ERK. Noteworthy, the amount of phosphorylated ERK to total ERK expression was diminished to a lower percentage in TNBC (9.1–35.5%) than in MCF7 (63.7%). Single treatment with the PD-1/PD-L1 inhibitor led to an increase of ERK phosphorylation in MCF7 and MDA-MB-231 cells with 154.3% and 190.2%, respectively, and a reduction to 48.6% in HCC1937 and did not regulate HCC1806. The combination of both inhibitors provoked a slight increase of ERK phosphorylation (116.8%) in MCF7 cells, but a distinct dephosphorylation in all TNBC cell lines (19.9% in MDA-MB-231, 65.2% in HCC1937 and 57.3% in HCC1806).

**Table 3:**
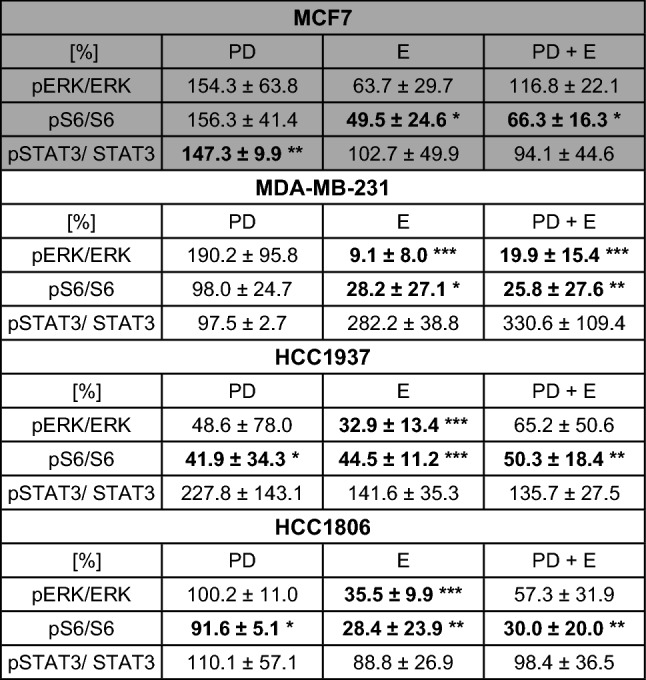
Ratios of pERK/ERK, pS6/S6, and pSTAT3/STAT3

Cells were treated with either 5 µM PD-1/PD-L1-Inhibitor 1 (PD), 5 µM ERK1/2 inhibitor (E) or the combination of both (PD + E) for 24 h. The percentage of phosphorylated to total protein was quantified considering protein loading amounts. Untreated cells set to 100% served as control). Results are presented as the mean ± standard deviation of three independent experiments in percent compared to the non-treated control [%]

Significant results in bold: *p < 0.05, **p < 0.01, ***p < 0.001 (*t* test)

The phosphorylation of S6 protein was enhanced after PD-1/PD-L1 inhibitor treatment in MCF7 cells, slightly decreased in MDA-MB-231 and HCC1806, and strongly reduced in HCC1937. In MCF7 the ERK1/2 inhibitor and the combined treatment significantly depleted S6 phosphorylation compared to untreated cells to 49.5 and 66.3%, respectively. In all TNBC cell lines, the ERK1/2 inhibitor and the combined treatment significantly deactivated S6 with the highest content in MDA-MB-231 with 28.3 and 25.8%, respectively.

The phosphorylation of STAT3 was strongly increased by PD-1/PD-L1 inhibitor treatment in MCF7 (147.3%) and in HCC1937 (227.8%) cells, but MDA-MB-231 and HCC1806 were nearly unchanged regarding STAT3 activation. The single ERK1/2 inhibitor and combined treatment showed no regulation of STAT3 in MCF7 and HCC1806 cells. However, in MDA-MB-231, the phosphorylation of STAT3 was increased to 282.2% and 330.6%, respectively, and in HCC1937 to 141.6% and 135.7%, respectively.

### Influence of PD-1/PD-L1 inhibitor and ERK1/2 inhibitor on gene expression of immune modulatory and growth-associated genes in TNBC and the non-TNBC cell lines

The effect of single and combined treatment of the PD-1/PD-L1 inhibitor and the ERK1/2 inhibitor on gene expression of the immune modulatory genes of PD-L1, IL-8, and CXCR2 as well as the growth-associated genes c-Fos and FosL was investigated in all cell lines after 24 h of treatment by qPCR.

The relative expression rates of PD-L1, IL-8, and CXCR2 after single and combined treatment are listed in Table 4: the PD-1/PD-L1 inhibitor did not regulate the PD-L1 gene in TNBC and only non-significant to 1.76-fold in MCF7. The ERK1/2 inhibitor significantly downregulated PD-L1 in MDA-MB-231 to 0.38 and non-significantly to 0.55 in HCC1806. In HCC1937 and MCF7, PD-L1 was upregulated about twofold. The combined treatment significantly increased PD-L1 gene expression up to 20 times in the basal-like cell lines HCC1937 and HCC1806, while in MDA-MB-231, PD-L1 expression was three times lower and in MCF7 1.7-fold higher. The IL-8 expression was significantly downregulated to its half amount in MDA-MB-231 by the PD-1/PD-L1 inhibitor while it was upregulated in the basal-like cell lines. The ERK1/2 inhibitor led to a significant upregulation of IL-8 expression both as single and combined treatment in MDA-MB-231 and HCC1806 cells. MCF7 cells showed a slight downregulation after PD-1/PD-L1 inhibitor treatment and an upregulation of IL-8 expression after ERK1/2 inhibitor and combined treatment. The CXCR2 expression was significantly downregulated by the combined treatment in MCF7, HCC1806 and MDA-MB-231, but it was upregulated in HCC1937.

**Table 4:**
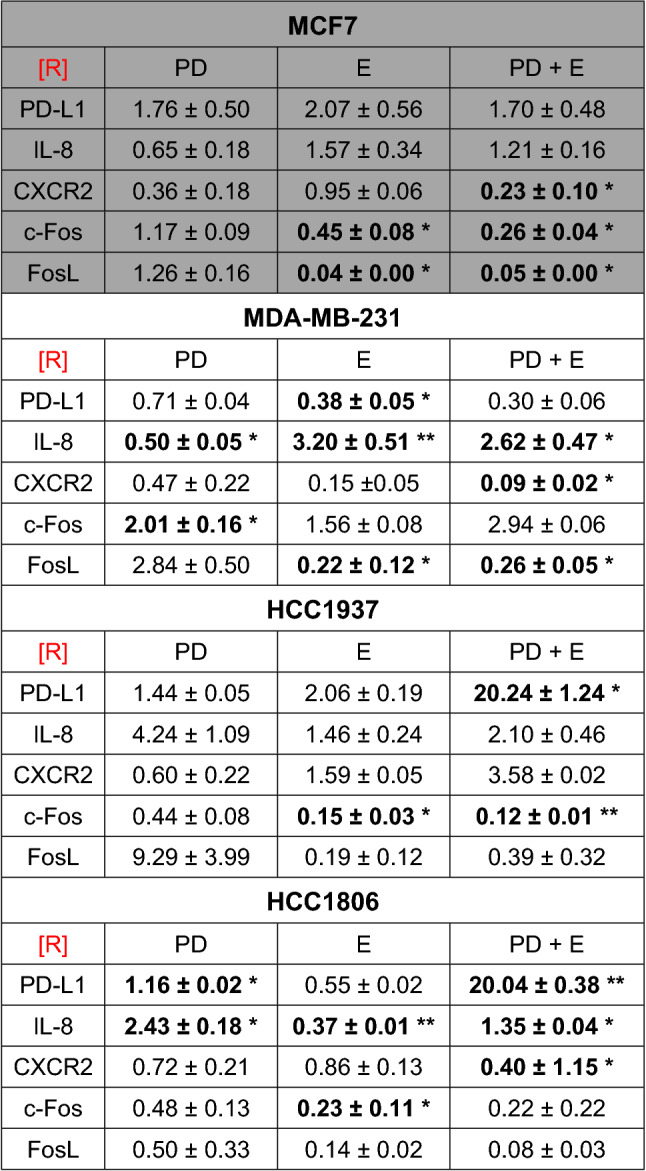
Regulation of immune modulatory and growth-associated genes after treatment with PD-1/PD-L1 and ERK1/2 inhibitors

Relative gene expression ratios [R] of PD-L1, IL-8, CXCR2, c-Fos and FosL in MCF7 (gray), MDA-MB-231, HCC1937, and HCC1806 (white) after treatment with PD-1/PD-L1 inhibitor 1 (PD), ERK1/2 inhibitor (E) and combined treatment (PD + E). Results are presented as the mean ± standard deviation of three independent experiments in relative gene expression ratios [R]

Significant results in bold: *p < 0.05, **p < 0.01 (*t* test)

C-Fos expression was downregulated in MCF7 and the basal-like cell lines by ERK1/2 inhibitor and combined treatment, while it was upregulated in MDA-MB-231 cells on trend. The expression of FosL was downregulated by ERK1/2 inhibitor and combined treatment in all investigated cell lines.

## Discussion

The application of immune checkpoint inhibitors in TNBC is subject of current cancer research. For a better response rate, combination treatment strategies with other antitumor agents were investigated in recent studies. Patients with TNBC response to anti-PD-1/PD-L1 therapy, and inhibitors of the MAPK/ERK signaling cascade showed impeding effect on proliferation and clinical potency in diverse cancer entities. Both signaling pathways, if activated, promote tumorigenesis on different molecular levels and therefore a combined blockage may be therapeutically promising.

In the present work, an anti-proliferative effect was confirmed on breast cancer cells by inhibitors of MAPK/ERK and PD-1/PD-L1 signaling pathways. Both inhibitors inhibited cell viability in all investigated breast cancer cell lines. Both, the PD-1/PD-L1 inhibitor as well as the ERK1/2 inhibitor, caused half-reduced cell viability (IC_50_) at lower concentrations in TNBC than in non-TNBC cell lines.

The TNBC cell lines and especially the group of basal-like subtypes, HCC1937 and HCC1806, seemed to react most sensitive to both inhibitors. In clinical studies, immune checkpoint inhibitors targeting PD-1/PD-L1 applied as single treatment regime reached response rates of 4.7 to 23.1% of patients of metastasized breast cancer dependent of subtype and PD-L1 expression. Within this group, TNBC patients with PD-L1-positive tumors frequently showed the highest objective response rates (Solinas et al. 2017). Adams and colleagues reported of response rates for pembrolizumab of 5.3% in TNBC (Adams et al. 2019b) and even 21.4% in PD-L1-positive TNBC (Adams et al. 2019a). In our work, a specific effect on immune response could not be investigated due to the missing PD-1 component in the in vitro tumor cell model. However, the PD-1/PD-L1 inhibitor seemed to perform a direct growth inhibition in TNBC cell lines. According to the work of Cui and coworkers, downregulation of PD-L1 with siRNA led to a decreased phosphorylation of ERK and STAT3 in oral squamous cell carcinoma cell lines. Furthermore, mesenchymal proteins involved in EMT were diminished in siPD-L1 treated cells (Cui et al. 2021) providing evidence that PD-L1 participates in proliferation and metastasis. An antitumoral effect by an inhibition of the MAPK signaling pathway in TNBC was confirmed by Nagaria and colleagues: the MEK inhibitor U0126 increased the cytotoxicity of the dual EGFR/HER2 inhibitor lapatinib additively, and of the Raf inhibitor sorafenib synergistically (Nagaria et al. 2017). Lee and colleagues demonstrated that the MEK inhibitor E6201 inhibited the colony formation rate, migration and invasion, caused cell cycle arrest, and induced apoptosis in TNBC cells (Lee et al. 2019b). In mice, E6201 inhibited tumor growth and lung metastasis, and improved survival (Lee et al. 2019b).

The treatment with the combination of PD-1/PD-L1 and ERK1/2 inhibitor achieved a synergistic inhibitory effect of proliferation in the basal-like and MCF7 cells and an additive effect in MDA-MB-231. Liu and colleagues investigated in a mouse model that combined immune checkpoint and MEK blockage led to a stronger growth inhibition in breast cancer compared to single treatment (Liu et al. 2015). Loi and coworkers showed in a TNBC mouse model that blocking the Ras/MAPK signaling pathway diminished the number of tumor-infiltrating T lymphocytes (TILs) and increased MHC and PD-L1 expression (Loi et al. 2016). This augmentation of antigen presentation that could lead to stronger immune response might be hindered by a PD-L1-induced T cell checkpoint blockage. So, a combination with a PD-L/PD-L1 inhibitor could sub-serve the immune response. Consistent with this, Loi and coworker reported of an increased anti-tumoral immune response achieved by combined treatment with different MEK and PD-1/PD-L1 inhibitors in TNBC (Loi et al. 2016). The COLET study resulted in a clinical benefit for patients with metastasized TNBC treated with advanced combination of cobimetinib, paclitaxel and atezolizumab (Brufsky et al. 2019). Thus, the combination of MEK and PD-1/PD-L1 inhibitors was frequently investigated and seemed to be effective, especially in TNBC.

Evidence of an effect of PD-1/PD-L1 and ERK inhibitors was proven by analyses of intracellular processes. As expected, the ERK1/2 inhibitor impeded the phosphorylation of ERK and S6 in our cell culture experiments. Giltnane and Balko reported a higher prevalence of activated MAPK signaling pathway in TNBC and basal-like breast cancer (BLBC) compared to other breast cancer subtypes (Giltnane and Balko 2014). Additionally, several working groups of Hoeflich (Hoeflich et al. 2009), Jing (Jing et al. 2012), and Mirzoeva (Mirzoeva et al. 2009) verified that MEK inhibitors particularly blocked proliferation in TNBC and BLBC. In the current work, the activation of ERK and S6 was clearly diminished by combined inhibitor treatment assuming that the proliferation inhibition occurred by impeded MAPK signaling pathways. Interestingly, in MCF7 cells, the combined treatment with both inhibitors displayed an increased phosphorylation of ERK. As a drawback, in our work, the PD-1/PD-L1 inhibitor activated ERK in MDA-MB-231 and MCF7. Black and coworker showed in breast cancer cell lines that resistance against chemo-therapeutics doxorubicin and docetaxel was increased by interaction of PD-1 and PD-L1 (Black et al. 2016). They also showed an increased activation of ERK in MDA-MB-231 by recombinant PD-1 inhibitor treatment (Black et al. 2016). However, in our study, the combined treatment with both inhibitors and the single treatment with the ERK inhibitor inhibited the ERK phosphorylation in MDA-MB-231 and the other TNBC cell lines. In the basal-like cell line HCC1937, ERK also was dephosphorylated by the PD-L1 inhibitor, only. This is in concordance to the downregulation of PD-L1 with siRNA in oral squamous cell carcinoma cell lines (Cui et al. 2021).

The oncogenes c-Fos and FosL were almost downregulated in TNBC by the combined treatment with both inhibitors. As part of the proliferation regulating transcription factor AP-1 (Saeki et al. 2009), c-Fos and FosL were described as oncogenes in different solid tumors e. g. in breast cancer (Lu et al. 2012). ERK activates the transcription of c-Fos (Saeki et al. 2009). In the present work, the ERK1/2 inhibitor reduced the c-Fos expression in all cell lines except in MDA-MB-231. FosL was reduced by all treatment including the ERK1/2 inhibitor. These reduced expressions of c-Fos and FosL might inhibit AP-1 and subsequently the proliferation. The PD-1/PD-L1 inhibitor as single treatment regulated the expression of FosL and c-Fos disparately, but did not rule over the effects of the combined treatment.

After treatment with the PD-1/PD-L1 inhibitor no or only a slight activation of STAT3 was found in TNBC or MCF7, respectively. On the other hand, Pardoll and coworkers reported of an activation of STAT3 that regulates the PD-L1 expression (Pardoll et al. 2012). Additionally, we detected a non-significant activation of STAT3 in TNBC and MCF7 cells after ERK1/2 inhibitor treatment. In contrast to our results, Sengupta and colleagues reported decreased phosphorylation of STAT3 by MEK inhibitor treatment (Sengupta et al. 1998). IL-8 gene expression is also activated by the treatments used here and IL-8 is able to initiate the phosphorylation of STAT3 (Ma et al. 2020). STAT3 activation is a critical point for the investigation of the treatment strategy of combined treatment with PD-1/PD-L1 and ERK1/2 inhibitor. Thus, it should be further investigated. Our results showed that the anti-proliferative effect by both inhibitors is concomitant with inhibition of the MAPK, but not with the STAT3 signaling pathway.

PD-L1 is discussed as predictive marker for treatment of breast cancer with immune checkpoint inhibitors. In our work, PD-L1 expression in HCC1937 and HCC1806 was significantly increased after combined treatment, but diminished in MDA-MB-231 and HCC1806 cells after single treatment with the ERK inhibitor. In concordance, Qian and colleagues showed a decreased PD-L1 mRNA expression after ERK1/2 inhibition in bladder cancer cells (Qian et al. 2008), and Liu and coworkers confirmed a downregulation of PD-L1 expression and ERK phosphorylation in NSCLC cells after PD0325901 or U0126 treatment (Liu et al. 2020). Several studies indicated an increased PD-L1 expression in TNBC compared to non-TNBC, though the results differed strongly (Barrett et al. 2015; Bedognetti et al. 2016; Gatalica et al. 2014). Furthermore, the definition of PD-L1 positivity varies. While some authors report that higher PD-L1 expression on tumor cells is associated with prognostic factors e. g. Ki-67 (Botti et al. 2017) and better disease prognosis (Barrett et al. 2018; Botti et al. 2017; Lee et al. 2019a), others showed the opposite (Choi et al. 2018; Tomioka et al. 2018; Zhu et al. 2019). The elevated PD-L1 expression after combined treatment in basal-like TNBC in the present work is difficult to assess, because we were unable to investigate the interaction with PD-1 and the tumor environment. A stronger expression of PD-L1 may be predictive for a better response rate to PD-1/PD-L1 therapy (Solinas et al. 2017).

Further immune modulatory genes were regulated after combined treatment of PD-L/PD-L1 and ERK1/2 inhibitor, e.g. IL-8 and its receptor CXCR2. IL-8 is involved in development and progress of TNBC (Hartman et al. 2013). The inhibition of IL-8 impairs migration and cell survival of TNBC cell lines (Fu and Lin, 2018). Therefore, the reduced expression of these genes may add to the efficiency of the tumor therapy. The downregulations of immune suppressing cytokines after inhibition of the Ras/MAPK/ERK signaling pathway were reported in different tumor cells of melanoma (Sumimoto et al. 2006), head and neck squamous cell carcinoma (Mohan et al. 2015) and breast cancer (Liu et al. 2017a, b). Here, IL-8 expression was significantly reduced in only the basal-like TNBC cell line HCC1806 after ERK1/2 inhibitor treatment. With the combination of PD-1/PD-L1 inhibition that blocks the immune modulation and the downregulation of IL-8 and its receptor CXCR2 by the ERK1/2 inhibitor, both medications could synergistically reanimate the immune system to target these TNBC subtypes. Kim and colleagues found significant higher expression of IL-8 in TNBC compared to non-TNBC which could be suppressed by the MEK inhibitor U0126 (Kim et al. 2016). Rody and coworkers observed that a lower IL-8 expression is accomplished with a better prognosis of TNBC patients (Rody et al. 2011). IL-8 promotes the secretion of growth factors by TILs (Waugh and Wilson, 2008), and thus, the proliferation of tumor cells. The expression of the CXCR2 receptor was significantly reduced by the combined treatment of PD-1/PD-L1 and ERK1/2 inhibitor in all investigated cell lines except the HCC1937. A meta-analysis of 4.012 patients with solid tumors showed a negative effect of an increased CXCR2 expression for the overall survival (Yang et al. 2017). Additionally, IL-8 is able to lead to chemotherapeutic resistance by autocrine secretion by its receptor CXCR2 (Al-Khalaf et al. 2019; Ha et al. 2017). The reduced CXCR2 expression after combined inhibitor treatment may improve prognosis of survival and counteract chemoresistance. It could be a drawback that the combination of ERK1/2 inhibitor with PD-1/PD-L1 inhibitor significantly increased IL-8 expression in MDA-MB-231 and HCC1806 in the present investigation. Possibly, the upregulation is clinically not relevant for the disease course. Overexpressed IL-8 may influence the activity of immune cells in the tumor micro-environment which has to be investigated.

Due to the different results among TNBC and non-TNBC cells as well as among TNBC subtypes, we found vague evidence, in which mechanisms caused proliferation inhibition in breast cancer cells by the PD-1/PD-L1 and ERK1/2 inhibitor. Of special interest was the question, if one inhibitor influences the signaling pathway or functional proteins of the other inhibitor, respectively, and prevent the appropriate effectiveness.

The PD-1/PD-L1 inhibitor did not essentially affect proteins of the MAPK signaling pathway. In TNBC cells, the ERK inhibitor activated STAT3 as signal protein for proliferation. Otherwise, the combination inactivated the signaling proteins ERK and S6 as well as the gene expression of c-Fos and FosL that strongly activates cell proliferation as part of the AP-1 complex. Consequently, we conclude that the combined use of PD-1/PD-L1 and ERK inhibitors may be a suitable option by the search for new therapy regimes of TNBC.

In cell culture experiments, the tumor micro-environment cannot be displayed as it is in vivo. Therefore, our investigation did not allow distinct conclusion for the clinical meaning of the combination of PD-1/PD-L1 and ERK inhibitor for breast cancer patients. Because of the importance of tumor micro-environment, e.g. the detection of cytotoxic (CD8 +) lymphocytes is predictive for the response to anti-PD-1 therapy (Tumeh et al. 2014), the next step has to be a cell culture model, where tumor cells are combined with immune cells. This could be realized in a co-culture setting with T cells, tumor-associated macrophages, and natural killer cells to simulate tumor environment.

## Data Availability

Data will be available and provided by request to the corresponding author.
